# Clinician perceptions of sleep problems, and their treatment, in patients with non-affective psychosis

**DOI:** 10.1080/17522439.2016.1206955

**Published:** 2016-07-29

**Authors:** Aliyah Rehman, Felicity Waite, Bryony Sheaves, Stephany Biello, Daniel Freeman, Andrew Gumley

**Affiliations:** ^a^ School of Psychology, University of Glasgow, Glasgow, Scotland; ^b^ Department of Psychiatry, University of Oxford, Warneford Hospital, Oxford, England; ^c^ Institute of Health and Wellbeing, University of Glasgow, Glasgow, Scotland

**Keywords:** Sleep, psychosis, schizophrenia, CBTi, clinicians

## Abstract

**Aims and method:** To assess clinicians’ views about their understanding and treatment of sleep problems in people with non-affective psychosis. An online survey was emailed to adult mental health teams in two NHS trusts.

**Results:** One hundred and eleven clinicians completed the survey. All clinicians reported disrupted sleep in their patients, and endorsed the view that sleep and psychotic experiences each exacerbate the other. However, most clinicians (*n* = 92, 82%) assessed sleep problems informally, rather than using standard assessment measures. There was infrequent use of the recommended cognitive-behavioural treatments for sleep problems such as persistent insomnia, with the approaches typically used being sleep hygiene and medications instead.

**Clinical implications:** Clinicians recognise the importance of sleep in psychosis, but the use of formal assessments and recommended treatments is limited. Barriers to treatment implementation identified by the clinicians related to services (e.g. lack of time), patients (e.g. their lifestyle) and environmental features of inpatient settings.

## Introduction

Sleep problems are a prominent concern for individuals with non-affective psychosis diagnoses such as schizophrenia, with estimates of the prevalence of sleep disturbance ranging between 30% and 80% (Cohrs, 2008). A range of sleep disorder problems have been reported in this patient group including insomnia (Cohrs, 2008), circadian rhythm disruption (Wulff, Dijk, Middleton, Foster, & Joyce, 2012), hypersomnia (Okruszek et al., 2014) and nightmares (Sheaves, Onwumere, Keen, Stahl, & Kuipers, 2015). There are numerous factors that may cause sleep problems in people with psychosis. A recent twin study found overlap in the genetic and environmental causes of both sleep disturbance and psychotic experiences such as paranoia (Taylor, Gregory, Freeman, & Ronald, 2015). Antipsychotic medications are known to interact with neurotransmitter systems that are involved in sleep/wake regulation and this may cause some sleep problems. For example, clozapine can increase sedation and aripiprazole has been linked to insomnia (Krystal, Goforth, & Roth, 2008). Psychotic experiences such as paranoia and voices may contribute to sleep disturbance (Jeppesen et al., 2015; Waite et al., 2015a). Finally, the high comorbidity of physical health problems in people diagnosed with schizophrenia may also contribute to poor sleep (Kalucy, Grunstein, Lambert, & Glozier, 2013). The view now stated in DSM-5 is that sleep problems should be assessed and treated irrespective of other psychiatric difficulties (American Psychiatric Association [APA], 2013). Clinical guidelines recommend hypnotics for acute insomnia and cognitive-behavioural therapy for persistent insomnia (CBTi) (Morin & Benca, 2012). There is emerging evidence that adapted CBTi can improve sleep in people with psychosis and may also lower levels of delusions and hallucinations (Myers, Startup, & Freeman, 2011). A pilot randomised clinical control trial with 50 patients with current delusions and hallucinations found that an eight-session CBTi intervention led to very large improvements in sleep (Cohens effect size 1.9, CI = 0.9, 2.9) (Freeman et al., 2015a). Adaptations for CBTi in people with psychosis have been described (Waite et al., 2015b), including a greater focus on circadian rhythm disruptions and the interaction with psychotic experiences. Furthermore, a recent qualitative study from this trial noted how highly valued the treatment of sleep problems is for this patient group (Waite et al., 2015a). However, the level of recognition in services of the extent of sleep problems in patients with psychosis and subsequent assessment and treatment in standard care has not been assessed. The aim of the current study was to address this gap.

## Methods

A 16-item online survey was delivered using Qualtrics survey software (Qualtrics, Provo, UT). As there was no suitable questionnaire available to assess clinician perceptions, we generated questions based on our own research and clinical experience. We wished to devise a questionnaire that would generate descriptive data that could inform future knowledge exchange aimed at facilitating discussion and debate about research and clinical priorities for clinicians and other stakeholders. A process of discussion and refinement lead to questions that spanned three areas of sleep: the prevalence/types of sleep problems (e.g. what are the most common complaints patients with psychosis report about their sleep?), the impact/causes (e.g. in your view, what causes the sleep problems in people who experience psychosis?) and the assessment/treatment (e.g. what methods do you use to treat sleep problems in patients with psychosis?). Furthermore, to gain more insight into what barriers currently exist in clinical practise, two open-ended questions were included: “In your view, are there any barriers to treating sleep problems in patients with psychosis?” “Are there any other comments you wish to add about sleep in your patients with psychosis?” The full list of questions is included in Appendix 1.

### Participants

Emails containing the survey link were sent to clinical teams treating patients experiencing psychosis in two NHS localities: Glasgow Greater Glasgow & Clyde and Oxford Health NHS Foundation Trust. The survey was also advertised in staff newsletters and on posters in staff rooms. The study was reviewed and approved by the University of Glasgow ethics committee (reference: 200,140,033).

The instructions displayed to clinicians on the survey included:

The questions in the survey ask about sleep in individuals with non-affective psychosis (e.g. schizophrenia, schizoaffective disorder and other non-affective psychotic disorders). Please answer the questions in relation to patients with non-affective psychosis.

### Data analysis

Descriptive statistics are presented for clinician responses to the questions. Reported percentages do not always add up to 100% because clinicians could select more than one answer for a number of the questions. The two open-ended questions were analysed qualitatively using the Framework Analysis approach (Ritchie & Spencer, 2002). Framework Analysis is a flexible approach to generating themes and organising different types of qualitative data (Ritchie & Spencer, 2002; Pope, Ziebland, & Mays, 2000). Analysis of clinician comments was conducted by AR, following the five steps outlined in Ritchie and Spencer (2002). These five steps were familiarisation with the data (reading and re-reading comments), developing themes (identifying key issues and concepts within the comments), indexing (linking the themes to the data), charting (rearranging data according to theme it fits with) and interpretation of the whole data set (linking the findings to the original research question). The credibility of the coding and thematic analysis was checked by AG.

## Results

### Clinician characteristics and type of service

One hundred and eleven clinicians completed the online survey including psychiatric nurses (*n* = 43, 39%), psychiatrists (38, 34%), psychologists (13, 12%), occupational therapists (7, 6%), social workers (6, 5%) and other (4, 4%). Almost half the clinicians were based in adult community mental health teams (CMHTs) (52, 47%). Other service types included early intervention services (22, 20%), inpatient wards (20, 18%), crisis service teams (1, 1%), primary care teams (1, 1%) and other (15, 13%).

### Prevalence and types of sleep problems

Sleep problems were considered to be very common in patients with psychosis, with all clinicians reporting sleep problems in their patients. Difficulties falling asleep (insomnia) and oversleeping (hypersomnia) were the most commonly reported sleep complaints, followed by issues with the timing of sleep (circadian rhythm disturbance). See Table 1 for the full description of the questions and responses.

**Table 1.** Clinician reports of sleep disorder prevalence rates and types of sleep disorders in patients with psychosis.

	0	1–20%	21–40%	41–60%	61–80%	81–100%	Unsure	
What percentage of patients that you see with psychosis who also have sleep problems? *N* (%)	0	*N* = 8 (7%)	*N* = 28 (25%)	*N* = 18 (16%)	*N* = 32, (29%)	*N* = 14 (13%)	*N* = 11 (10%)	
	Insomnia	Sleep-related movement disorder	Sleep-related breathing disorder	Hypersomnia	Circadian rhythm disruption	Narcolepsy	Parasomnias	Other
What are the most common complaints patients with psychosis report about their sleep? (Select all that apply)	*N* = 97 (87%)	*N* = 28 (25%)	*N* = 7 (6.3%)	*N* = 79 (71%)	*N* = 57 (51%)	*N* = 2 (2%)	*N* = 30 (27%)	*N* = 2 (2%)

Note:

Please note that the numbers do not add up to 100% because multiple answers could be selected.

### Impact/causes of sleep problems

Sleep problems were viewed to have a negative impact in a wide range of domains with nearly all (104, 93%) clinicians reporting that sleep problems have a negative impact on daytime functioning. No clinician endorsed the item “there is no link between sleep and psychosis”. The majority of clinicians (104, 93%) endorsed the option that the relationship between sleep problems and psychosis is bidirectional (i.e. that both sleep and psychotic symptoms influence each other).

Various causes were reported to cause sleep problems in people with psychosis. Poor sleep hygiene was the most commonly reported and negative symptoms was the least common. A number of clinicians (18, 16%) stated other causal factors (e.g. lack of daytime activity, smoking and illicit drug use). See Table 2 for a full description of the questions and responses.

**Table 2.** Clinician reports of the impact and causes of sleep problems in patients with psychosis.

	Mood (e.g. anxiety)	Positive Symptoms (.e.g. paranoia)	Negative Symptoms (e.g. anhedonia)	Cognitive Functioning (e.g. attention)	Physical Health (e.g. feeling unwell)	Social Functioning (e.g. relationships)	Daytime Activities (e.g. attending appointments)
In your view, do the sleeping problems that patients with psychosis experience have a negative impact in any of the following domains? (Select all that apply)	*N* = 101 (91%)	*N* = 78 (70%)	*N* = 79 (71%)	*N* = 93 (84%)	*N* = 89 (80%)	*N* = 96 (87%)	*N* = 104 (94%)
	Sleep problems are one factor that can make psychotic symptoms (positive and/or negative) worse	Psychotic symptoms (positive and/or negative) are one factor that can make sleep worse	Both are true (the relationship between sleep and psychosis is bi-directional)	Unsure	Sleep and psychosis are not related		
What is your understanding of the relationship between sleep and psychosis?	*N* = 2 (2%)	*N* = 3 (3%)	*N* = 104 (93%)	*N* = 2 (2%)	*N* = 0 (0%)		
	The sleep problems are a consequence of medication	The sleep problems are a result of poor sleep hygiene (e.g. too much caffeine, napping)	The sleep problems are a consequence of affective symptoms (e.g. low mood, anxiety)	The sleep problems are a consequence of positive psychotic symptoms	The sleep problems are a consequence of negative psychotic symptoms	Other	
In your view, what causes the sleep problems in people who experience psychosis? (Select all that apply)	*N* = 64 (58%)	*N* = 87 (79%)	*N* = 71 (64%)	*N* = 79 (71%)	*N* = 61 (55%)	*N* = 18 (16%)	

Note:

Please note that the numbers do not add up to 100% because multiple answers could be selected.

### Assessment/treatment

#### How often do you formally assess sleep problems in people with psychosis?

Over half of clinicians (60, 54%) were unsure about how often they were formally assessing sleep problems. Twenty-three (21%) reported assessing sleep problems in the majority of their patients, 13 (12%) reported assessing sleep in around half of their patients, 11 (10%) reported assessing sleep in a minority of their patients and four (3%) reported assessing sleep rarely or never.

#### What methods do you use to assess sleep problems in people with psychosis?

The majority of clinicians (92, 83%) reported assessing sleep by informally asking patients, followed by 18 (16%) using structured interviews, four (4%) using self-report tools and two (2%) using objective methods (e.g. actigraphy). Twelve clinicians (11%) used “other” methods of assessment which included observations in wards, sleep diaries and verification from family/friends.

#### What methods do you use to treat sleep problems in patients with psychosis?

A range of treatment methods were used by clinicians with the most common being sleep hygiene (104, 94%), and CBTi was least frequently endorsed (13, 12% delivering the intervention; 19, 17% referring for CBTi intervention). Medication was commonly used for treating sleep problems: hypnotics (64, 58%), antipsychotics (49, 44%), antidepressants (42, 38%) and anxiolytics (30, 27%).

#### What form of sleep treatment would you recommend for a patient with long-standing sleep difficulties and psychotic symptoms?

Fifty-seven (51%) clinicians recommended multiple sleep treatments including medication, sleep hygiene, online self-help and CBTi. A number of clinicians recommended a single treatment: 45 (40%) recommended sleep hygiene, 27 (24%) recommended medication, 24 (22%) recommended CBTi and 16 (14%) recommended online self-help.

### Qualitative analysis

From the qualitative analysis, three themes emerged regarding barriers to treating sleep problems in people with psychosis. The first theme was termed “Patient-related factors” which was further split into two subthemes: (i) “Lifestyle of the patient” and (ii) “Illness-related factors”. “Lifestyle of the patient” included aspects of the patient’s life that can act as a barrier to treatment such as lack of routine, alcohol/drug dependence, lifestyle habits, social support, individual differences and motivation levels. The second subtheme, “Illness-related factors”, included severity of disorder, stage of illness, affective disturbance, capability of patient and medication and their side effects. The second theme was termed “Service-related factors”, which included lack of time, lack of awareness of sleep as a problem, resources, access to treatments and training needs. We also noted that a number of clinicians perceived there to be no barriers to treating sleep problems. The third theme was termed “Other environmental factors”, and this included barriers that are found on wards and in prisons. Examples from each theme are presented in Table 3.

**Table 3.** Qualitative themes with example quotations.

Theme one: Patient-related factors		Theme 2: Service-related factors	Theme 2: Other environmental factors
Subtheme 1: Lifestyle of patient	Subtheme 2: Illness-related factors		
“Lifestyles can also contribute to poor sleep hygiene and patients may have a reluctance to engaging in work to improve daily structure”	“Patient too unwell to follow treatment and advice, too anxious to follow any plan”	“Time pressure”	“Chaotic and noisy wards”
“Sleep problems more likely where there is alcohol or substance misuse or dependence”	“Medication side effects – particularly sedation leading to poor sleep patterns”	“Lack of knowledge in staff”	“Inpatients have trouble sleeping because hospital wards are not conducive to good sleep”
“Motivation and willingness to do the work necessary”	“Patients may be reluctant to engage, suspicious, or fearful of the process”	“Lack of knowledge and resources to treat sleep problems”	“In prisons, there is a reluctance to prescribe sedative medication because of its ‘street value’ amongst prisoners – makes treating sleep problems in the prison (a very big problem) difficult”
	“In an acute state I think you have to take a medication route before even thinking about CBT for insomnia”	“Clinicians may not assess complaints of sleep problems in sufficient detail”	“Lack of therapeutic interventions on ward”
		“It appears to me to be a significant issue that often seems neglected or viewed as a symptom of mental distress”	

## Discussion

This is the first study to ask clinicians about their perceptions of sleep in people with non-affective psychosis. There was a clear disconnect: the importance of sleep in psychosis was well-recognised, but assessment and treatment were limited. Clinicians also reported a number of barriers to treatment implementation.

We found that clinicians were highly aware of the range of sleep problems that present in people with psychosis, particularly insomnia, hypersomnia and circadian rhythm disturbance. Clinicians also identified a link between poor sleep and several domains of functioning including level of symptoms, mood and daytime activities. There is some support for these observations, with poor sleep being associated with lower quality of life (Hofstetter, Lysaker, & Mayeda, 2005), increased positive symptoms (Afonso, Brissos, Figueira, & Paiva, 2011) and impaired cognitive functioning in people with psychosis (Bromundt et al., 2011). Additionally, previous research has found links between sleep disturbance and impaired physical health and mood disturbance (Taylor et al., 2007, Cappuccio, Cooper, D’Elia, Strazzullo, & Miller, 2011; De Wild-Hartmann et al., 2013). These findings suggest that not only are sleep problems common and diverse, but that poor sleep may be negatively affecting many areas of functioning in people with psychosis. This emphasises the importance of treating sleep problems in this population.

Clinicians also reported multiple causes of the sleep problems including poor sleep hygiene, positive symptoms, affective symptoms, followed by medications and negative symptoms. Work on the causes of sleep problems in people with psychosis is currently underway. There is growing evidence to suggest that sleep problems may precede the onset of psychosis, with a recent review concluding that sleep disturbance is very common in individuals at risk for psychosis (Davies, Haddock, Yung, Mulligan, & Kyle, in press). For example, in one longitudinal follow-up study in people at risk for developing psychosis, sleep disturbance predicted transition to psychosis (Ruhrmann et al., 2010). Another line of work has examined the relationship between sleep disturbance and individual psychotic experiences (Reeve, Sheaves, & Freeman, 2015). For example, in a large-scale general population study conducted in 8580 people, insomnia was associated with an approximately two- to threefold increase in paranoid thinking (Freeman et al., 2010). A follow-up study reported that insomnia was also a significant predictor of new incidences of paranoid thoughts, suggestive of insomnia having a causal role (Freeman et al., 2012). The OASIS trial is currently underway testing the potential causal relationship between insomnia, paranoia and hallucinations (Freeman et al., 2015b). Other additional causal factors identified were also reported as barriers to treating sleep problems in people with psychosis such as lack of routine, low levels of daytime activity and drug/alcohol dependence. Another barrier recognised by some clinicians concerned medications and their side effects. Although antipsychotics are the recommended treatment for psychosis and such medications tend to improve sleep continuity (Cohrs, 2008; Monti & Monti, 2004), their side effects such as sedation may cause further sleep problems.

Clinicians mainly assessed sleep complaints in people with psychosis by informally asking them about their sleep. Few used objective (e.g. actigraphy) and subjective methods (questionnaires), although a number of clinicians (*n* = 10, 15%) used structured interviews to assess sleep. Patients’ own informal accounts of sleep problems are a quick and easy way to gain an impression of sleep problems when clinical time is limited. However, an in-depth and accurate assessment of sleep problems is critical in identifying the most appropriate treatment pathway that can target the specific sleep problem. Clinicians identified time as a barrier to treating sleep problems. This barrier could be addressed by making available brief and validated questionnaires that can assess the severity of the sleep problem and allow monitoring of sleep over time such as the Sleep Condition Indicator and the Epworth Sleepiness Scale (Espie et al., 2014; Johns, 1991). The Sleep Condition Indicator has been developed specifically for clinicians to assess insomnia quickly and accurately. The short two-item version would be a particularly useful initial screening tool to assess insomnia severity in people with psychosis and sleep problems (Espie et al., 2014).

We also found that clinicians were treating sleep problems mainly using sleep hygiene techniques, despite the limited effectiveness of such approaches (Morgenthaler et al., 2006). There was also reliance on medications such as hypnotics, which should only be used for short periods of acute insomnia (Morin & Benca, 2012). Critically, we found that even though clinicians recommended a range of treatments including CBTi, the numbers of clinicians actually referring people with psychosis to such services is much lower. According to recent guidelines, CBTi is the recommended treatment for persistent insomnia and the use of medications for other psychiatric disorders is not currently recommended (APA, 2013; Morin & Benca, 2012). Some of the barriers identified by clinicians may explain the discrepancy between recommendation of CBTi and low referral rates such as lack of access to sleep treatments. This barrier could be addressed by making CBTi more widely accessible. Other barriers reported by clinicians include those specific to patients such as low motivation to change habits. These are barriers that can be addressed by highlighting the importance of good sleep, and empowering people with psychosis to gain control of their own sleep using evidence-based techniques (Waite et al., 2015a).

Finally, clinicians also noted that barriers to treating sleep problems exist in very specific environments such as inpatient wards and prisons. For example, assessing and treating sleep problems may be difficult in wards due to noise levels, night-time observations and limited resources to implement interventions. More work is required to address these barriers.

The findings of our study should be viewed in light of some limitations. First, clinicians who are interested in sleep would be more aware of sleep problems and may have been more motivated to complete the survey, a source of bias in our sample. The survey was also completed mainly by psychiatrists and psychiatric nurses and completed from clinicians in only two NHS trusts, one of which was the site for an intervention study for sleep in patients with psychosis. Despite this, the results of our survey are clear: sleep problems are considered common in people with psychosis, but services are currently limited in their responses to this issue.

### Clinical implications and recommendations for future studies

Developing treatment pathways is an important clinical aim. Greater awareness about the importance of sleep among both staff and people with psychosis is also required. Training programmes would enable staff to identify, assess and treat sleep problems. We call for more research on understanding the causes of sleep problems in non-affective psychosis and testing the implementation of evidence-based sleep interventions in patients. More work is also required to identify the impact of different medications on sleep.

## Disclosure statement

No potential conflict of interest was reported by the authors.

## Funding

This work was supported by Economic and Social Research Council grant No. [ES/J500136/1]; Wellcome Trust grant No. [09846/Z/12/Z].

## Appendix 1.

**Table:**

Question(s)	Answer options
What is your job title?	Choose from: Psychiatrist, Psychiatric Nurse, Psychologist, OT, Social Worker, Primary Care Team, or Other
What type of service are you based in?	Choose from: Inpatient Ward, Adult Community Health Team, Early Intervention Services, Crisis Resolution Team, Primary Care Team or Other
Where are you based?	Choose from: Oxford, Glasgow, Birmingham, Manchester, Edinburgh, Liverpool
What percentage of patients that you see with psychosis, also have sleep problems?	Choose from:
	−0%
	−1–20%
	−21–40%
	−41–60%
	−61–80%
	−81–100%
What are the most common complaints patients with psychosis report about their sleep? (Select all that apply)	(More than one option can be selected)
	• Insomnia (difficulties getting to sleep or staying asleep)
	• Sleep-related movement disorders (e.g. restless legs syndrome, periodic limb movements)
	• Sleep-related breathing problems (e.g. sleep apnoea)
	• Hypersomnia (sleeping for very long periods, feeling tired in the day)
	• Circadian rhythm disruption (e.g. mistimed sleep: going to bed too early, or very late)
	• Narcolepsy
	• Parasomnias (e.g. nightmares or sleepwalking)
	• Other
In your view, do the sleeping problems that patients with psychosis experience have a negative impact in any of the following domains? (Select all that apply)	(More than one option can be selected)
	• Mood (e.g. anxiety) • Positive symptoms (e.g. paranoia) • Negative symptoms (e.g. anhedonia) • Cognitive functioning (e.g. attention) • Physical health (e.g. feeling unwell) • Social functioning (e.g. relationships) • Daytime activities (e.g. attending appointments)
In your view, what causes the sleep problems in people who experience psychosis? (Select all that apply)	(More than one option can be selected)
	• The sleep problems are a consequence of medication • The sleep problems are a result of poor sleep hygiene (e.g. too much caffeine, napping) • The sleep problems are a consequence of affective symptoms (e.g. low mood, anxiety) • The sleep problems are a consequence of positive psychotic symptoms • The sleep problems are a consequence of negative psychotic symptoms • Other
What is your understanding of the relationship between sleep and psychosis?	• Sleep problems are one factor that can make psychotic symptoms (positive and/or negative) worse
	• Psychotic symptoms (positive and/or negative) are one factor that can make sleep worse
	• Both of the above are true (it works both ways)
	• Unsure
	• Sleep and psychosis are not related
How often do you formally assess sleep problems in people with psychosis?	• Rarely/never
	• In a minority of cases
	• In around a half of cases
	• In the majority of cases (75%)
	• In nearly all or all patients with psychotic symptoms
What methods do you use to assess sleep problems in people with psychosis? (Select all that apply)	• Informally ask patient
	• Objectively assess sleep (e.g. EEG, neck circumference)
	• Psychometric self-report (e.g. administer the Insomnia Severity Index questionnaire)
	• Structured interview assessment
	• Other
How often are you treating sleep problems in people with psychosis?	• Rarely/never
	• In a minority of cases
	• In around a half of cases
	• In the majority of cases (75%)
	• In nearly all or all patients with psychotic symptoms
What methods do you use to treat sleep problems in patients with psychosis? (Select all that apply)	Choose from:
	• Antipsychotics
	• Hypnotics
	• Anxiolytics
	• Antidepressants
	• Refer for cognitive-behavioral therapy for insomnia
	• Deliver cognitive-behavioural therapy for insomnia
	• Sleep hygiene tips
	• Offer psychoeducation materials
	• There are no effective treatments for sleep problems in people with psychosis
	• Other
In your view, are there any barriers to treating sleep problems in patients with psychosis?	Open-ended answer
What do you think could be the benefits of improving sleep in people who experience psychosis? (select all that apply)	• None
	• Improved psychotic symptoms
	• Improved affective symptoms
	• -Increased energy
	• -Increased engagement in activities
	• -Improved physical health
	• -Other
What form of sleep treatment would you recommend for a patient with long-standing sleep difficulties and psychotic symptoms? (Select all that apply)	• -Medication
	• -Sleep hygiene tips (from a leaflet or website)
	• -Online self-help
	• -Cognitive-behavioural therapy for insomnia
	• All of the above
Are there any other comments you wish to add about sleep in your patients with psychosis?	Open-ended answer
